# Targeting CD47 as a Novel Immunotherapy for Multiple Myeloma

**DOI:** 10.3390/cancers12020305

**Published:** 2020-01-28

**Authors:** Jennifer Sun, Barbara Muz, Kinan Alhallak, Matea Markovic, Shannon Gurley, Zhe Wang, Nicole Guenthner, Katherine Wasden, Mark Fiala, Justin King, Daniel Kohnen, Noha Nabil Salama, Ravi Vij, Abdel Kareem Azab

**Affiliations:** 1 Department of Radiation Oncology, Cancer Biology Division, Washington University in St. Louis School of Medicine, St. Louis, MO 63108, USA; jennifer.sun@wustl.edu (J.S.); bmuz@wustl.edu (B.M.); kinanalhallak@wustl.edu (K.A.); sgurley@wustl.edu (S.G.); wangzhe@wustl.edu (Z.W.); nicole.guenthner@wustl.edu (N.G.); k.e.wasden@wustl.edu (K.W.); 2 Department of Biomedical Engineering, Washington University in St. Louis McKelvey School of Engineering, St. Louis, MO 63130, USA; 3 Department of Pharmaceutical and Administrative Sciences, St. Louis College of Pharmacy, St. Louis, MO 63110, USA; matea.markovic@stlcop.edu (M.M.); noha.salama@stlcop.edu (N.N.S.); 4 Department of Medicine, Oncology Division, Washington University in St. Louis School of Medicine, St. Louis, MO 63110, USA; mfiala@wustl.edu (M.F.); justinking@wustl.edu (J.K.); daniel.kohnen@wustl.edu (D.K.); rvij@wustl.edu (R.V.); 5 Department of Pharmaceutics and Industrial Pharmacy, Faculty of Pharmacy, Cairo University, Cairo 11562, Egypt

**Keywords:** multiple myeloma, macrophages, checkpoint inhibitors, 3D tissue culture model

## Abstract

Multiple myeloma (MM) remains to be incurable despite recent therapeutic advances. CD47, an immune checkpoint known as the “don’t eat me” signal, is highly expressed on the surface of various cancers, allowing cancer cells to send inhibitory signals to macrophages and impede phagocytosis and immune response. In this study, we hypothesized that blocking the “don’t eat me” signaling using an anti-CD47 monoclonal antibody will induce killing of MM cells. We report that CD47 expression was directly correlated with stage of the disease, from normal to MGUS to MM. Moreover, MM cells had remarkably higher CD47 expression than other cell populations in the bone marrow. These findings indicate that CD47 is specifically expressed on MM and can be used as a potential therapeutic target. Further, blocking of CD47 using an anti-CD47 antibody induced immediate activation of macrophages, which resulted in induction of phagocytosis and killing of MM cells in the 3D-tissue engineered bone marrow model, as early as 4 hours. These results suggest that macrophage checkpoint immunotherapy by blocking the CD47 “don’t eat me” signal is a novel and promising strategy for the treatment of MM, providing a basis for additional studies to validate these effects in vivo and in patients.

## 1. Introduction

Multiple myeloma (MM) is a cancer of plasma cells in the bone marrow (BM) and represents the second most common hematologic malignancy in the world [1]. In the past decade, therapeutic breakthroughs such as proteasome inhibitor (PIs), immunomodulatory drugs (IMiDs), and antibody-based therapeutics have substantially expanded the number of treatment regimens available for patients in all stages of MM [2]. However, despite the recent advances, MM remains to be incurable because almost all patients eventually relapse or become refractory to treatment, which lowers the median survival to only 5–9 months [3]. Therefore, new approaches are needed to effectively target and eliminate MM.

Recently, cancer immunotherapy has gained heightened attention as a promising approach for treatment of MM and relapse/refractory MM, since many tumor-associated antigens have been identified in MM cells [4]. Immunotherapies focus on the repair, stimulation, and/or enhancement of the body’s natural immune responses to fight cancer. The recovery of immune surveillance can block tumor development with fewer adverse effects, which can serve as a powerful tool for long-term control of MM.

Among the most promising approaches for activation of antitumor immunity is immune checkpoint blockade. Immune checkpoints are inhibitory pathways that help keep immune responses “in check” and prevent immune cells from killing normal cells, hence also referred as the “don’t kill me” signal [5]. However, cancer cells are found to overexpress immune checkpoint proteins on the surface [6], making them less visible to immune surveillance [7,8]. Blocking these checkpoints on cancer cells effectively releases the “brakes” on the immune system, allowing for a restored antitumor immune response [9]. Examples of checkpoints that negatively regulate T-cell immune functions include programmed cell death protein 1 (PD-1; on T cells) and its ligand (PD-L1; on target cells), as well as cytotoxic T-lymphocyte-associated antigen 4 (CTLA-4; on T cells) and its ligands (B7-1/B7-2, on target cells) [10]. Immune checkpoint blockade using monoclonal antibodies (mAbs) as inhibitors against these targets has become a paradigm-shifting treatment in solid tumors and blood cancers, enabling patients to produce an effective anti-tumor response [11]. However, this strategy have not shown capacity for effectiveness in MM; MM monotherapy of PD-1/PD-L1 inhibitors showed unsatisfactory clinical results [12,13]. Therefore, alternative strategies such as checkpoint proteins for other immune cell types are being explored.

Macrophages are part of the innate immune system, and they represent the first line of defense and respond quickly to threats such as tissue damage or infection [14]. Specifically, macrophages are the “professional eaters” of the immune system specialized in the detection, phagocytosis, and destruction of foreign substances, microbes, cancer cells, and other harmful organisms [15]. In addition, macrophages also function as antigen presenting cells, which induce and direct adaptive immune response (such as in T cells and B cells) [16].

Similar to T cells, macrophages also express a checkpoint receptor called signal regulatory protein α (SIRPα, also known as CD172a), which recognizes the surface receptor CD47 as ligand on target cells. The interaction between SIRPα and CD47 initiates a signaling cascade that results in the inhibition of macrophage phagocytic activity, hence referred as the “don’t eat me” signal [17]. CD47 has been shown to commonly overexpress on cancer cells, including hematologic malignancies [18,19,20] and numerous solid cancers [21].

Currently there are a wide range of studies aiming to inhibit the CD47-SIRPα immune checkpoint using various strategies, including anti-CD47 antibodies, anti-SIRPα antibodies, and soluble SIRPα proteins [22]. The best characterized therapies targeting this checkpoint are anti-CD47 antibodies, which has proven effective in inducing phagocytosis of tumor cells in vitro as well as inhibiting tumor growth in mice models of both hematologic and solid tumors [23,24]. Additionally, there are multiple phase I/II clinical investigations on the therapeutic efficacy of anti-CD47 antibodies on hematologic and solid malignancies as single agent or combination treatment [17]. However, there are limited number of studies on targeting CD47 in MM, while prior results from other types of cancers suggest encouraging outlook for similar strategies in treating MM.

The aim of this study is to investigate the effect of a new anti-CD47 antibody Vx1000R on inducing phagocytosis and killing of MM cells. We hypothesized that blocking CD47 on MM cells with mAbs will enhance phagocytosis and killing of MM, which represents a novel strategy for MM cancer immunotherapy.

## 2. Results

### 2.1. CD47 Expression in MM Patients

**Figure 1:**
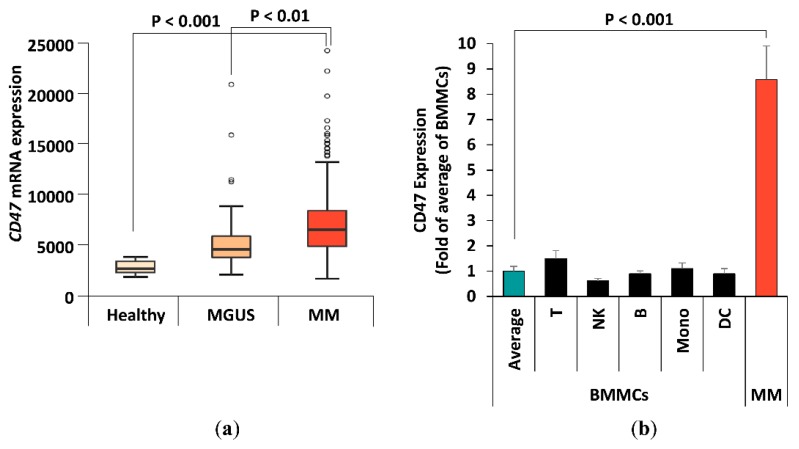
CD47 expression in multiple myeloma (MM) patients. (**a**) CD47 mRNA expression level in CD138+ bone marrow plasma cells from healthy subjects (*n* = 22), MGUS (*n* = 44), and newly diagnosed MM patients (*n* = 559). (**b**) CD47 protein expression of subpopulations in MM patient BM samples (*n* = 4). Subpopulations include CD3 (T cells), CD14 (monocytes/macrophages), CD16 (natural killer cells-NKs, eosinophils, and neutrophils), CD19 (B cells), CD123 (dendritic cells-DCs and basophils), and CD138 (MM cells).

The aim of this study is to investigate the effect of a new anti-CD47 antibody Vx1000R on inducing phagocytosis and killing of MM cells. We hypothesized that blocking CD47 on MM cells with mAbs will enhance phagocytosis and killing of MM, which represents a novel strategy for MM cancer immunotherapy. First, we compared the *CD47* gene expression of BM CD138+ plasma cells between MM subjects of different disease stages using datasets published on Gene Expression Omnibus by Zhan and Shaughnessey [25]. We analyzed *CD47* mRNA expression for patients of three stages: healthy (*n* = 22), monoclonal gammopathy of undetermined significance (MGUS; a premalignant stage of MM) (*n* = 44), and newly diagnosed MM (*n* = 559) (Figure 1a). It can be appreciated that *CD47* mRNA expression markedly increases in accordance with disease progression, suggesting it being a potential prognostic marker for MM. More importantly, *CD47* is highly expressed in newly diagnosed MM patients, making anti-CD47 mAbs a desirable treatment strategy.

Next, we analyzed the expression of CD47 protein in malignant plasma cells as well as immune cell populations in MM patient samples. BM mononuclear cells (BMMCs) were isolated from patient BM aspirates (*n* = 4) obtained from Washington University in St. Louis Medical School. CD47 protein expression in BMMCs samples were analyzed by Vx1000R mAb binding. Various sub-populations were identified by labeling their CD markers with respective antibodies. These populations included CD3 (T cells), CD14 (monocytes/macrophages), CD16 (NK cells, eosinophils, neutrophils), CD19 (B cells), CD123 (DCs and basophils), and CD138 (MM cells). Flow cytometry analysis shows CD47 protein to be ubiquitously expressed on all cell population tested, but especially high in CD138+ MM cells (Figure 1b). CD138+ cells showed 8.5-fold higher CD47 expression comparing to the average of other mononuclear populations shown (*p* < 0.001).

### 2.2. The Effect of Tumor Microenvironment on CD47 Expression in Cell Lines

We also tested CD47 expression in three human (MM.1S, H929, U266) and one mouse (5TGM1) MM cell lines frequently used in the laboratory to determine if they are good models for in vitro investigation. The expression was evaluated through flow cytometry via Vx1000R binding (Figure S1). Myeloma cell lines were shown to display high levels of CD47 in a universal manner (Figure S2), similar to the levels observed in the primary patient samples.

**Figure 2:**
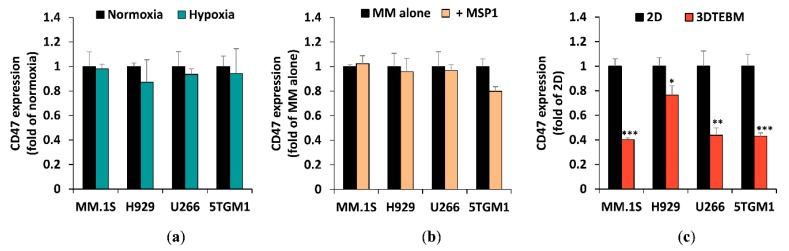
CD47 expression in human (MM.1S, H929, U266) and mouse (5TGM1) MM cell lines under different culture conditions. (**a**) CD47 protein expression in hypoxia (1% O_2_) as fold of normoxic (21% O_2_) condition. (**b**) The effect of co-culture with MM-associated stromal cell line MSP-1 on CD47 expression. (**c**) The effect of 3DTEBM culture on CD47 expression compared to classic 2D culture. (* *p* < 0.05; ** *p* < 0.01; *** *p* < 0.001).

Then we tested the effect of the tumor microenvironment (TME) on CD47 expression in MM. Previously, hypoxia has been shown to be a general feature of many hematologic malignancies, including MM. Specifically, hypoxia was shown to be a driving factor for MM metastasis and was heavily involved in cancer drug resistance [26,27]. We tested the effect of hypoxia on the expression of CD47 on the surface of MM cells, and found that MM cell lines conserved their CD47 expression under hypoxic conditions (Figure 2a). Another important feature of MM TME is the stroma, known to play an important role in processes such as differentiation, migration, proliferation, survival, and drug resistance [28]. Previously, our lab has established a myeloma-derived stromal cell line named MSP-1 [29]. It was shown that MSP-1 affected proliferation, adhesion, migration, and drug resistance in MM cells in a more profound manner than healthy stromal cell lines. We tested the effect of co-culturing MM cells with myeloma-derived stromal cells MSP-1 on expression of CD47, and found that MM did not induce significant change in CD47 expression levels (Figure 2b). In addition to the 2D classic tissue culture models, we tested a more patho-physiologically relevant 3D culture model (3D tissue engineered bone marrow, 3DTEBM) on the expression of CD47 in MM cells [28]. When we cultured the cell lines in 3DTEBM, their expression of CD47 were downregulated two- to three-folds (Figure 2c).

### 2.3. Effect of Vx1000R on MM Killing in 2D and 3DTEBM

**Figure 3:**
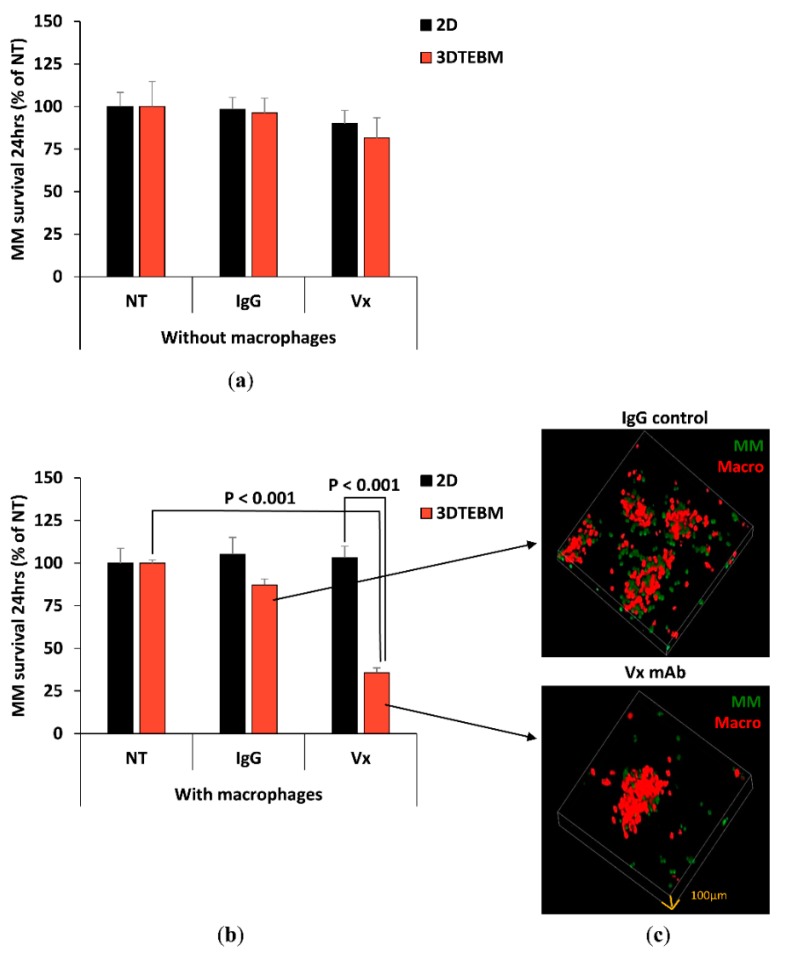
Effect of Vx1000R on MM killing. (**a**) MM cell survival in NT, IgG control, and Vx1000R (5 ug/mL) conditions at 24 h in 2D vs. 3DTEBM cultures without macrophages, represented as % of NT. (**b**) MM cell survival in NT, IgG control, and Vx1000R (5 ug/mL) conditions at 24 h in 2D vs. 3DTEBM cultures with macrophage co-culture, represented as % of NT. (**c**) Representative confocal z-stack images of 3DTEBM co-cultures at 24 h, treated with IgG control (top) or Vx1000R mAb (bottom).

We then investigated the effect of anti-CD47 mAb Vx1000R as a therapeutic antibody. We treated MM cells with Vx1000R or its IgG isotype control in classic 2D cultures or in the 3DTEBM, and looked at the antibody’s capacity for MM killing without the presence of macrophage. We found that neither the IgG control nor the Vx1000R induced cytotoxicity to MM cells, without the presence of macrophages (Figure 3a).

Then we tested the effect of IgG and Vx1000R on MM survival in the presence of macrophages, in 2D and 3DTEBM. In the 2D tissue culture model, neither IgG nor Vx1000R had an effect on the killing of MM. In contrast, in the 3DTEBM, inhibition of CD47 with Vx1000R induced significant killing of MM (25% survival) compared to no-treatment control or the IgG isotype antibody (Figure 3b). We also saw that the killing effect in 3DTEBM started as early as 4 hours and continued over 24 h (Figure S3). To further visualize MM killing by CD47 inhibition in 3DTEBM, we imaged the MM-macrophage 3DTEBM co-cultures treated with IgG control or with Vx1000R. Consistent with the quantitative data from the flow cytometry analysis, confocal imaging showed less MM cells in the culture treated with Vx1000R compared to the culture treated with IgG control (Figure 3c).

### 2.4. Effect of Vx1000R on Phagocytosis of MM by Macrophages

**Figure 4:**
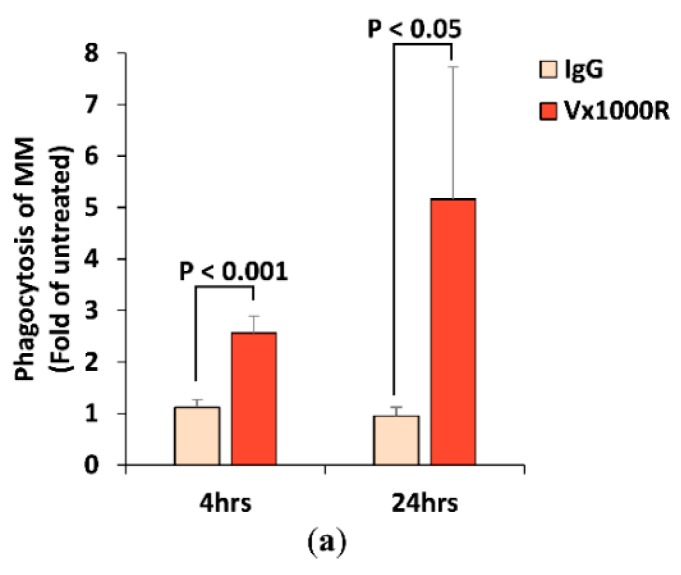

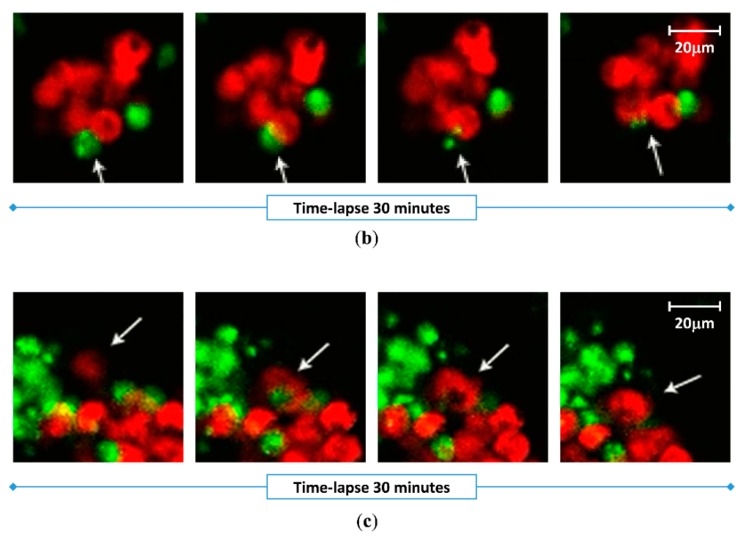
Effect of Vx1000R on phagocytosis. (**a**) Phagocytosis in 3DTEBM co-cultures at 4 h or 24 h, as fold of untreated condition. (**b**,**c**) Real-time live confocal imaging capturing MM phagocytosis by macrophages.

We further studied the effect of IgG and Vx1000R on MM phagocytosis by macrophages. We found that IgG did not alter the phagocytosis of MM; however, Vx1000R induced phagocytosis as early as 4 h and continued to increase over 24 h (Figure 4a). To further visualize the effect of CD47 inhibition on induction of phagocytosis, we imaged the MM-macrophage 3DTEBM co-cultures treated with Vx1000R using real-time live confocal imaging and captured time-lapse videos showing macrophages engulfing and digesting MM cells (Figure 4b,c and Videos S1–S3).

## 3. Discussion

The use of checkpoint inhibitors to increase the T-cell activity has shown very promising clinical results in different solid tumors [30,31,32,33]. However, similar strategies have not shown capacity for effectiveness in MM; MM monotherapy of PD-1/PD-L1 inhibitors showed unsatisfactory clinical results [12,13]. These outcomes can be attributed to immunosuppression often seen in many MM patients, where impaired T cells characterized by exhaustion and senescence were detected in the BM [34]. On the contrary, levels of monocytes/macrophages were found to be elevated in the BM of MM patients [35]. Therefore, in this study, we proposed checkpoint inhibition of macrophages as a potential treatment for MM.

First, we explored the expression of CD47 in MM patients. We found that the gene expression of CD47 was directly correlated with stage of the disease. Specifically, plasma cells from MM patients overexpress CD47 compared to those from MGUS patients, which had a higher expression compared to the normal subjects. In addition, in patient BMMCs, the MM cell population had a remarkably higher protein expression of CD47 than other cell populations. These findings indicated that CD47 is specifically expressed and can be a potential target for the treatment of MM.

Additionally, we found that the expression of CD47 on MM cell lines was universally high, which is in agreement with previously published information [24]. We also studied the effects of different factors (such as hypoxia or co-culture with stroma) on the expression of CD47 in MM cells. Both hypoxia and stroma were previously shown to change the expression of MM surface biomarkers and sensitivity to therapy [29,36,37]. We found that neither of these had an effect on CD47 expression. Surprisingly, we found that when cultured in the 3DTEBM, MM cells show a downregulated expression of CD47. Our lab has developed the 3DTEBM model derived from the BM of MM patients, which demonstrated a much closer model to the patho-physiology of the MM BM niche, and allowed proliferation of primary MM cells ex vivo, and recapitulated the clinical drug sensitivity/resistance profile of the MM cells ex vivo [28].

As a therapeutic approach, we tested the effect of blocking CD47 “don’t eat me” signal on the killing of MM using an anti-CD47 mAb (Vx1000R). We first tested the effect of the anti-CD47 mAb on MM survival in the absence of macrophages, and found that it did not induce any killing of MM cells. However, when MM cells were treated and a co-cultured with macrophages, significant killing of MM cells was seen in the 3DTEBM, but not in classic 2D cultures. The classical 2D culture model lacks the complexity to accurately describe the complex biology of MM and drug responses observed in patients, which often leads to obstacles when it comes to clinical translation.

Previously, it has been shown that myeloma TME contains elevated levels of CD47 ligands [38]. Binding of CD47 surface receptor to its ligands induces endocytosis of the ligand-receptor complex, which results in the removal of CD47 from cell surface [39]. Therefore, the myeloma microenvironment which exists in 3DTEBM cultures may have contributed to the CD47 downregulation seen in 3DTEBM compared to 2D culture models. Additionally, fibrin- and collagen- based 3D cultures are known to support higher cell motility compared to cell adherent to tissue culture plastics [40]. Supplementary videos show extensive motility of macrophages during the phagocytosis process, which is allowed because of the hydrogel-like structure of the 3DTEBM, similar to the conditions in vivo, and in contrast to the adherent nature of the 2D cultures of macrophages. These two factors may explain the enhanced phagocytosis and killing of MM in the 3DTEBM compared to regular 2D tissue culture models.

It is important to note that checkpoint inhibition in T cells takes a few days to demonstrate killing of MM cells in vitro [41,42], in this study however, we demonstrated that macrophages were effective immediately, and that the killing of MM cells was observed as early as 4 h. We further validated that this effect was, indeed, mediated by phagocytosis of MM cells by macrophages.

## 4. Materials and Methods

### 4.1. Cell Culture

#### 4.1.1. MM Cell Lines

Human MM cell lines (MM.1S, H929, and U266) were purchased from the American Type Culture Collection (ATCC, Rockville, MD, USA). The murine MM cell line 5TGM1-GFP-Luc was a kind gift from Dr. John DiPersio (Washington University School of Medicine, St. Louis, MO, USA). MM cells were cultured with RPMI-1640 media (Sigma-Aldrich, St. Louis, MO, USA) supplemented with 10% fetal bovine serum (FBS; Gibco, Life Technologies, Grand Island, NY, USA), 1% L-Glutamine, and 1% Penicillin-Streptomycin (Corning, Tewksbury, MA, USA). Cells were cultured at 37 °C and in 5% CO_2_ in a NuAire water jacket incubator (normoxia, 21% O_2_) (NuAire, Plymouth, MN, USA) or in hypoxic chamber (hypoxia, 1% O_2_) (Coy, Grass Lake, MI, USA). Media were refreshed every 3–4 days. MM.1S, H929, and 5TGM1-GFP cells were removed from flasks via gentle scrapping. Myeloma-derived stromal cell line MSP-1 was previously established by our lab [29]. MSP-1 was cultured with DMEM media (Sigma-Aldrich) with the same supplements as above, and removed with 0.25% trypsin (Corning) for up to 2 min.

#### 4.1.2. Mice BM Macrophages (BMMs)

Primary mice-derived macrophages were differentiated from the BM of SCID mice (Wilmington, MA, USA). Approval for these studies was obtained from the Ethical Committee for Animal Experiments at Washington University in St. Louis School of Medicine (protocol code: 20180263). Briefly, mice femurs were isolated and flushed with phosphate buffered saline (PBS; Sigma-Aldrich) to obtain the BM. Marrow cells were then filtered through a 70-μm filter to create a single cell suspension as well as to remove any unwanted hair, bone chips, etc. They are then spun down and cultured in 6-well plates with RPMI medium enriched with 10% FBS, 1% L-glutamine, 1% penicillin-streptomycin, and 25 ng/mL macrophage colony-stimulating factor (Mouse M-CSF; Sino Biological, Beijing, China) or 10% L929-conditioned medium. Non adherent cells were removed 1 day after initial plating and media were subsequently refreshed every 3 days following thorough PBS washing. BMMs are differentiated for 7 days and are defined by protrusions and adherence. BMMs can be lifted from the wells using 0.25% trypsin (Corning) or Accutase (Sigma-Aldrich).

### 4.2. Gene Expression

Gene expression of *CD47* (Probe ID 213857_s_at) in CD138 selected BM plasma cells was compared in newly diagnosed MM patients (*n* = 559), MGUS patients (*n* = 44), and healthy subjects (*n* = 22); data were obtained from Gene Expression Omnibus database available online (GSE2658 and GSE5900) [25]. Analysis and visualization were done in SAS (version 9.3). ANOVA and Tukey’s range test were used for comparisons between the three subject groups.

### 4.3. Protein Expression by Flow Cytometry

#### 4.3.1. Protein Expression in MM Primary Patient Cells

Mononuclear cells from the BM (BMMCs, *n* = 4) of MM patients were obtained from the Siteman Cancer Center, Washington University School of Medicine in St. Louis, MO. Informed consent was obtained from all patients with an approval from the Washington University in St. Louis School of Medicine Institutional Review Board Committee (protocol code: 201102270) and in accordance with the Declaration of Helsinki. Samples were isolated by red cell lysis, as previously described [37]. BMMCs were first stained with Vx1000R or isotype-control mAb at 5 µg/mL for 1 h at 4 °C. Vx1000R anti-CD47 mAb and its isotype control were provided by Vasculox Inc. (St. Louis, MO, USA). The cells were then washed thoroughly with PBS, and stained with a secondary goat-anti-mouse AF633 secondary antibody (Thermo Fisher Scientific, Waltham, MA, USA) for 1 hour at 4 °C. Afterwards, samples were washed and divided into six, and each was stained with FITC-labeled antibodies against CD3 (T cells), CD14 (monocytes/macrophages), CD16 (natural killer cells-NKs, eosinophils, and neutrophils), CD19 (B cells), CD123 (dendritic cells-DCs and basophils), or CD138 (MM cells). FITC antibodies for flow cytometry analysis were obtained from Miltenyi Biotec (Auburn, CA, USA). Flow cytometry analysis was carried out using MACS Quant Analyzer 10 Flow Cytometer (Miltenyi Biotec). Each subpopulation of BMMCs (T cells, monocytes, NKs, B cells, CDs and MM) was gated as FITC+ cells, and the CD47 expression of each population was demonstrated as the relative mean fluorescence intensity (RMFI) of AF633 for Vx1000R as fold of isotype-control.

#### 4.3.2. Cell Lines

MM cell lines (MM.1S, H929, U266, and 5TGM1) were cultured in normoxia (21% O_2_) or hypoxia (1% O_2_) in classic (2D) tissue culture plates. MM cell lines were stained with Calcein AM and co-cultured with MSP-1 stromal cell line in 2D cultures. Additionally, Calcein AM labeled MM cells were cultured in the 3DTEBM that contained all the cellular fraction of the BM isolated from MM patients. At 72 h, cells were retrieved, washed, and stained with Vx1000R or isotype-control mAb, followed by AF633 secondary antibody, and analyzed by flow cytometry.

### 4.4. 3DTEBM Culture

3D-Tissue Engineered BM (3DTEBM) cultures (Cellatrix, St. Louis, MO, USA) were established by crosslinking fibrinogen in patient BM supernatant using CaCl_2_ (Sigma-Aldrich), as previously described [28]. The 3DTEBM culture is a 3D scaffold obtained by crosslinking of fibrinogen found in patient bone marrow supernatant into fibrin, which makes the scaffold. The culture’s cellular content can be customized by inclusion of various cell populations. For investigating CD47 expression in MM cells (Section 4.3.), the full cellular fraction of the BM microenvironment was used. For testing the role of macrophages in the efficacy of the anti-CD47 antibody (Section 4.5 and Section 4.6), MM cells were cultured with or without macrophages only. The 3DTEBM scaffolds were supplemented with media on top and incubated at 37 °C. At time of analysis, the scaffolds were digested with collagenase (Gibco, Life Technologies) for 1.5–2 h at 37 °C; cells were retrieved, washed, and subjected to flow cytometry analysis.

### 4.5. Cell Survival and Phagocytosis by Flow Cytometry

5TGM1-GFP+ MM cells treated with or without IgG isotype control or Vx1000R (5 µg/mL) were cultured alone, or co-cultured with DiD-labeled BMMs for 24 h in classic 2D cultures or in the 3DTEBM culture. MM cells were retrieved from the cultures by trypsinization of the 2D culture or digestion of the 3DTEBM culture using collagenase. MM cell survival was determined as the count of GFP+ cells per replicate, normalized to counting beads (Invitrogen, Carlsbad, CA, USA), and demonstrated as percent of untreated. Phagocytosis was determined as percent of GFP and DiD double positive cells, and demonstrated as fold of untreated.

### 4.6. Cell Survival and Phagocytosis by Confocal Microscopy

5TGM1-GFP MM cells were co-cultured with DiD-labeled BMMs and treated with or without IgG isotype control or Vx1000R (5 μg/mL) in 3DTEBM, plated in a Nunc™ Lab-Tek™ II 8-well chamber slide (Thermo Fisher Scientific). Cultures were placed on the confocal microscope stage with isolated environment of 37 °C and 5% CO_2_. Cultures were imaged live at 2 h to detect phagocytosis by taking images from the green channel (for MM) and the red channel (for BMMs) using a 40× lens on a Zeiss LSM 510 meta confocal microscope (Zeiss, Oberkochen, Germany). Time lapse videos were taken for 90 min every 3 min per frame. Additionally, Z-stacks of the whole culture were created at 24 h using a 10× lens at 100 µm height with 3.5 µm step size, to confirm the killing effect detected by flow cytometry.

### 4.7. Statistical Analysis

All experiments were performed in at least triplicates, and cell line experiments were repeated at least three times. Results were expressed as means ± standard deviation, and statistical significance was analyzed using a Student’s t-test or one-way ANOVA. P values less than 0.05 were used to indicate statistically significant differences.

## 5. Conclusions

In summary, we have shown that (1) CD47 mRNA expression correlates with disease progression, (2) CD47 expression is profoundly higher in myeloma cells compared to other populations in patient PBMCs, (3) this expression was downregulated in 3DTEBM cultures, and (4) blocking CD47 on MM cells with anti-CD47 mAb enhanced MM killing by macrophages especially in 3DTEBM, which was mediated by an enhanced phagocytosis. Our findings suggest that macrophage checkpoint inhibition by blocking the CD47 “don’t eat me” signal is a novel and promising immunotherapy for the treatment of MM, and provides a basis for additional studies to validate the use of targeting CD47 in vivo and in patients.

## Supplementary Materials

The following are available online at https://www.mdpi.com/2072-6694/12/2/305/s1. Figure S1: Vx1000R binding curve on MM.1S cell line. Figure S2: CD47 protein expression of MM cell lines under 2D normoxic (21% O_2_) condition. Figure S3: The effect of Vx1000R treatment in MM survival in MM-macrophage co-cultures at 4 h and 24 h in (**a**) 2D and (**b**) 3DTEBM cultures. Video S1: Representative live confocal time-lapse video of phagocytosis in 3DTEBM co-cultures of untreated condition. Videos S2 and S3: Representative live confocal time-lapse video of phagocytosis in 3DTEBM co-cultures of Vx1000R (5 ug/mL) treated conditions.
