# Supplementary material for: Targeting CD47 as a Novel Immunotherapy for Multiple Myeloma
Source: Cancers (Basel). 2020 Jan 28;12(2):305. doi: 10.3390/cancers12020305 (PMC7072283; doi:10.3390/cancers12020305)
Supplement: Supplementary file 1 [file cancers-12-00305-s001.zip › cancers-685865-final/Supplementary Figures.pdf]

Supplementary Figures

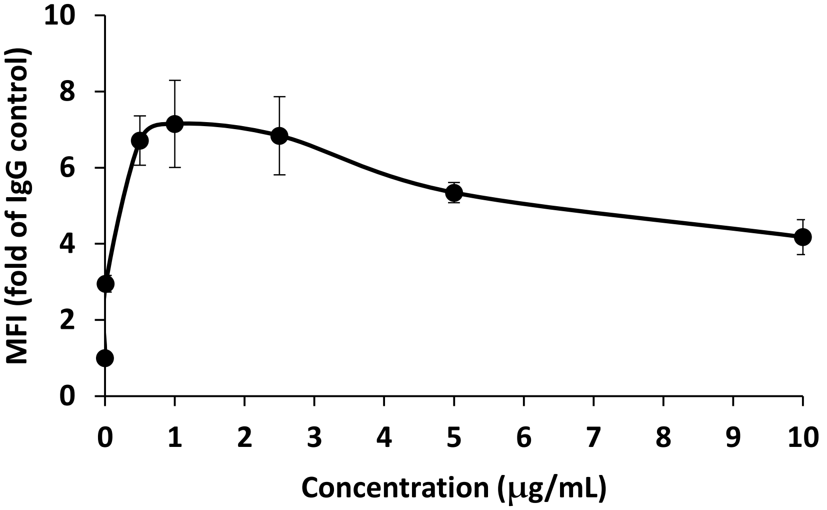

Figure S1. Vx1000R binding curve on MM.1S cell line.

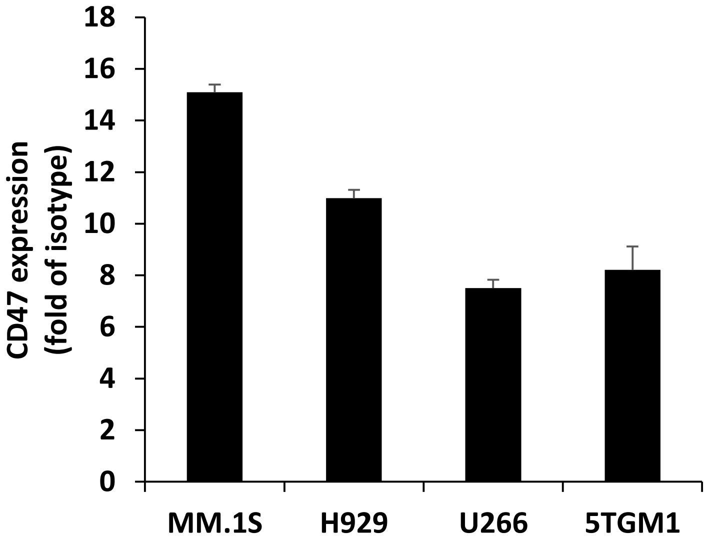

Figure S2. CD47 protein expression of MM cell lines under 2D normoxic (21% O_2) condition.

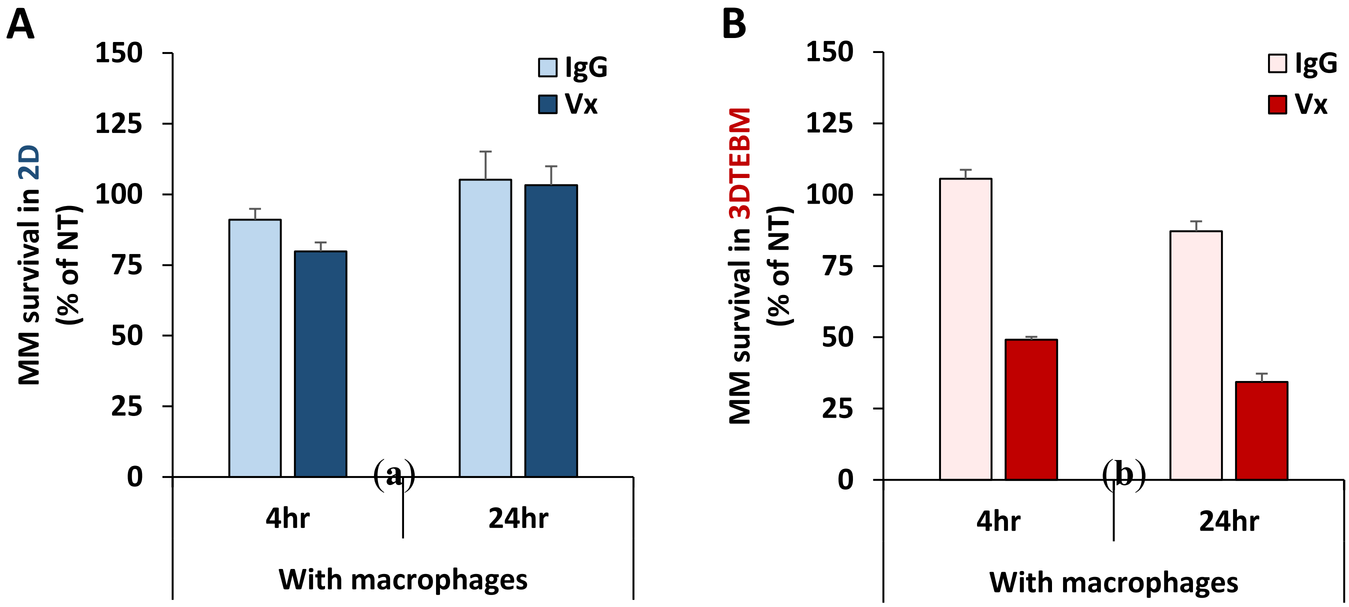

Figure S3. The effect of Vx1000R treatment in MM survival in MM-macrophage co-cultures at 4 h and 24 h in (**a**) 2D and (**b**) 3DTEBM cultures.
